# A Computational Protocol Combining DFT and Cheminformatics for Prediction of pH-Dependent Redox Potentials

**DOI:** 10.3390/molecules26133978

**Published:** 2021-06-29

**Authors:** Rocco Peter Fornari, Piotr de Silva

**Affiliations:** Department of Energy Conversion and Storage, Technical University of Denmark, Anker Engelunds Vej 301, 2800 Kongens Lyngby, Denmark; rocfor@dtu.dk

**Keywords:** redox flow batteries, high-throughput screening, computational protocol, redox potential prediction, quinones, solvation free energy

## Abstract

Discovering new materials for energy storage requires reliable and efficient protocols for predicting key properties of unknown compounds. In the context of the search for new organic electrolytes for redox flow batteries, we present and validate a robust procedure to calculate the redox potentials of organic molecules at any pH value, using widely available quantum chemistry and cheminformatics methods. Using a consistent experimental data set for validation, we explore and compare a few different methods for calculating reaction free energies, the treatment of solvation, and the effect of pH on redox potentials. We find that the B3LYP hybrid functional with the COSMO solvation method, in conjunction with thermal contributions evaluated from BLYP gas-phase harmonic frequencies, yields a good prediction of pH = 0 redox potentials at a moderate computational cost. To predict how the potentials are affected by pH, we propose an improved version of the Alberty-Legendre transform that allows the construction of a more realistic Pourbaix diagram by taking into account how the protonation state changes with pH.

## 1. Introduction

**Scheme 1:**
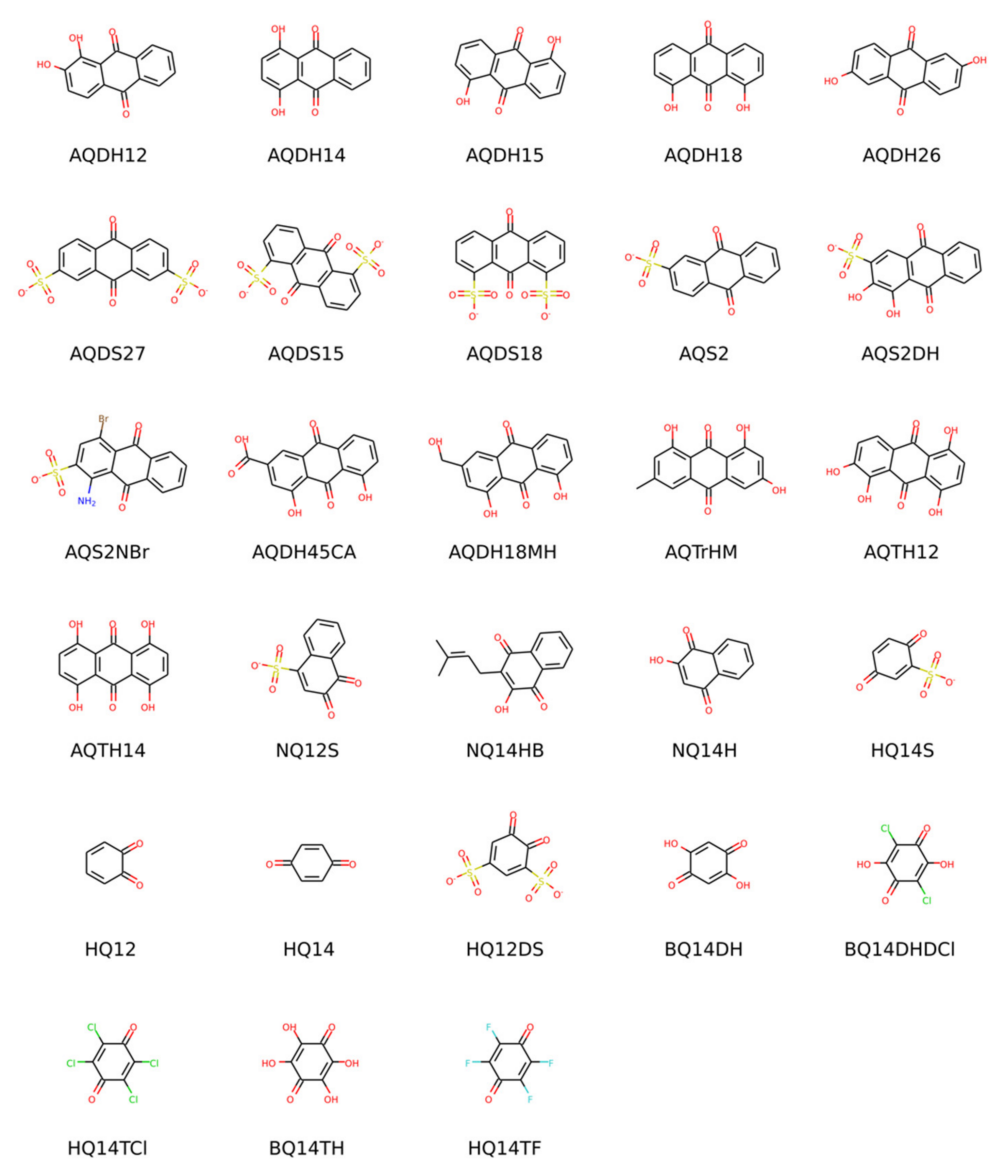
Structures and abbreviations of the molecules considered in this study in their oxidized form and most prevalent protonation state at pH = 0.

The main goal of this contribution is the establishment and validation of a standard procedure for the computational prediction of redox potentials of organic molecules undergoing a proton-coupled electron transfer. One of the main motivations for benchmarking such predictions is the computational screening of the vast chemical space of organic molecules to identify electrolytes for redox flow batteries (RFBs) [1,2,3,4,5,6,7,8,9,10,11,12,13,14]. At some point in any computational workflow for materials discovery, one needs a reliable method to predict with reasonable accuracy and moderate computational cost the properties of an already pre-selected candidate pool. The redox potential is one of the most important properties of redox-active materials. Its pH dependence is especially important in aqueous RFBs, where the pH affects the molecules’ solubility and electrochemical behavior and can even be exploited to increase the voltage [15]. The main theoretical concepts and the most commonly used computational methods for modeling organic redox materials have recently been summarized by the present authors in a review article [16]. Here, we will not repeat those general concepts and focus instead on how different methods and details of the procedure affect the agreement with experimental data. We will use notation consistent with [16] throughout this article. The experimental redox potentials at pH 0, 7, and 13 reported by Wedege et al. [17] for a set of 28 molecules of the quinone family have been chosen as a consistent data set for validating our computational protocol. It includes molecules with a different number of aromatic rings (i.e., benzo-, naphtho- and anthraquinones) and a diverse range of substituents and therefore span a wide range of redox potentials. Their structures are shown in Scheme 1 and full names in Table S1 in the Supplementary Materials. Although we validate the procedure on quinones, it is generalizable to other classes of redox molecules regardless of the number of exchanged electrons and/or protons.

## 2. Computational Protocol and Results

The procedure we present is designed to be integrated into a computational discovery workflow: it takes as input the molecular structures of the oxidized and reduced forms (Ox and Red), typically in a text string format such as SMILES, and outputs redox potentials as a function of pH. It can briefly be summarized as follows: (1) determine the main protonation state at pH 0 and find the lowest energy conformer of Ox and Red, (2) calculate the free energies of Ox and Red to obtain the standard redox potential $U^{0}$, (3) transform $U^{0}$ to the pH values of interest.

In proton-coupled redox reactions, H^+^ is one of the reagents. $U^{0}$ is defined in the thermodynamic standard state where [H^+^] = 1 M or pH = 0. Redox potentials at other pH values, i.e., at conditions different from the standard state, are described by the Nernst equation [18]. The pH of the solution also determines the protonation state of the reactants and products, and therefore affects their energies and the number of protons exchanged in the redox reaction. The redox potential depends essentially on the reaction-free energy in the solution (see Equations (1) and (2)). In principle, the redox potential at any pH could be calculated from the free energies at the protonation state most abundant at that pH. However, at high pH, most OH groups tend to be deprotonated so that the major species of hydroxylated reduced quinones can have several negative charges (up to 6 in the set considered here). Such multiple anions can be challenging for the convergence of electronic structure methods since the additional electrons may not be sufficiently stabilized [16]. Moreover, the accuracy of implicit solvation models is known to deteriorate when increasing the charge of the solute [19]. For these reasons, it is preferable to compute the redox potential at pH = 0 and then transform it to higher pH values [20,21] using expressions based on the Nernst equation, such as Equation (5).

### 2.1. Initial Guess: Protonation State and Conformer

Step 1 of the procedure outlined above, whose goal is to determine the most likely molecular structure as an initial guess for the subsequent geometry optimization, is performed with inexpensive cheminformatics and force-field-based tools. For each SMILES string, the protonation state of the major microspecies at pH = 0 is determined using the pK_a_ calculator of the cheminformatics software package ChemAxon [22]. The resulting SMILES string of the pH = 0 structure is then converted to a 3D structure using Open Babel, [23,24] and its lowest energy conformation is determined using a conformer search script, part of the AMS software suite [25], which is based on RDKit [26] and was locally modified by us to use the MMFF94 force field [27] instead of UFF. This modification was necessary because UFF does not correctly identify the lowest energy conformers with intramolecular hydrogen bonds, while MMFF94 does. This is due to a different treatment of electrostatic interactions (the main component of hydrogen bonding) in the two force fields [28,29]. The relationship between intramolecular hydrogen bonding and stability has been reported before [10,30].

### 2.2. Calculation of the Redox Potential at pH 0

For a general proton-coupled reduction reaction $\mathrm{Ox}+ne^{-}+n_{p}H^{+}\to \mathrm{Red}$ where n electrons and $n_{p}$ protons are transferred to the molecule, the standard redox potential is:

(1) $$U^{0}=-\frac{1}{ne}\Delta G_{\mathrm{sol}}^{0}-U_{\mathrm{SHE}},$$

where *n* is the number of electrons exchanged, *e* is the elementary charge and $U_{\mathrm{SHE}}$ = 4.43 V [31] is the absolute potential of the standard hydrogen electrode. The reduction free energy in solution $\Delta G_{\mathrm{sol}}^{0}$ (expressed in eV) can be written as

(2) $$\Delta G_{\mathrm{sol}}^{0}=G_{\mathrm{sol}}^{0}\left(\mathrm{Red}\right)-G_{\mathrm{sol}}^{0}\left(\mathrm{Ox}\right)+n_{p}\Delta G_{s}^{0}\left(H^{+}\right),$$

i.e., the difference between the free energies of solvated Red and Ox, balanced by the solvation free energy of $n_{p}$ protons, where $\Delta G_{s}^{0}\left(H^{+}\right)=-11.38\;\mathrm{eV}$ [32]. The accuracy of the calculated redox potential depends essentially on the electronic structure methods and approximations adopted to compute the free energies in solution. Our goal is to determine which combination of methods performs best against experimental reference data.

#### 2.2.1. Choice of Standard Method

The most common choice for computing molecular properties at a quantum chemical level is density functional theory (DFT), as it offers reasonably high accuracy at a moderate computational cost. We choose as a standard method the B3LYP hybrid density functional, a very popular choice as a general-purpose method for ground-state geometry optimizations. The DFT-D3-BJ dispersion correction [33] is always included unless stated otherwise. We use the ADZP basis set (double zeta with polarization and diffuse functions) as it is a good compromise between size and cost for molecular properties. All DFT calculations were performed with the Amsterdam Density Functional (ADF) software version 2019.302 [34,35].

#### 2.2.2. Solvation and Thermal Contributions to Free Energy

Quantum chemical methods are often used in conjunction with implicit solvation models to include the effect of the solvent on the molecule’s free energy. Here, we use the COSMO [36,37] implicit solvation model as a standard method. The geometry optimization can be performed directly in solution (with COSMO), or a single point COSMO calculation can be performed at the geometry optimized in the gas phase. The thermal contribution to the free energy, which includes zero-point energy, vibrational enthalpy, and entropy, is obtained from a harmonic vibrational frequency calculation in the gas phase at the BLYP/ADZP level, which is much faster than B3LYP since ADF cannot compute analytical gradients with hybrid functionals. This frequency calculation is done on a geometry optimized with the same method (BLYP in the gas phase).

**Figure 1:**
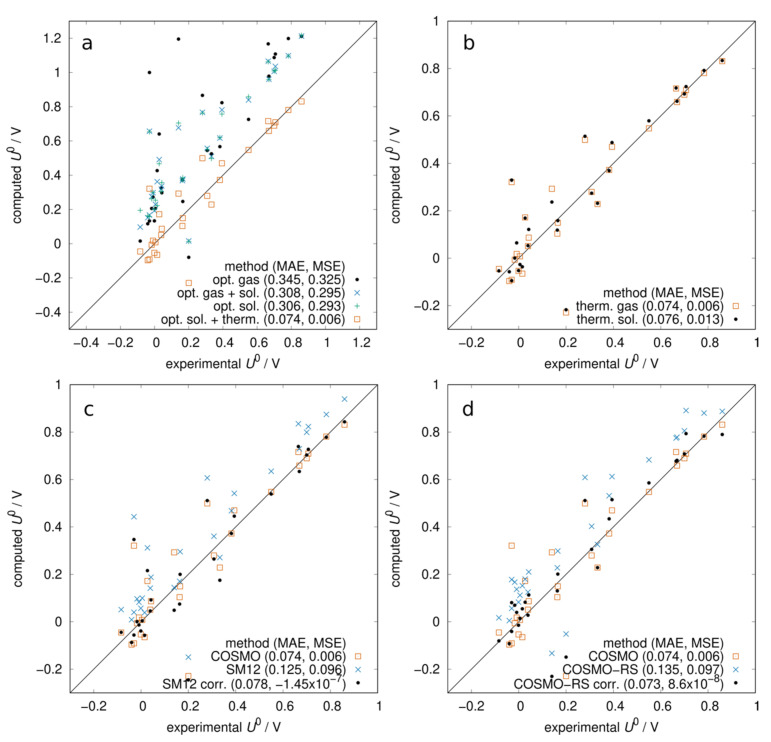
Computed vs. experimental redox potentials at pH = 0: role of solvation and thermal contributions to the free energy. All energies computed with B3LYP functional and ADZP basis set. (**a**) Starting from gas-phase electronic energies, the effect of adding solvation (COSMO) and thermal contribution (from BLYP gas-phase frequencies). (**b**) Comparison between thermal contribution computed in gas or solution. (**c**) Comparison between COSMO and SM12 solvation models. SM12 calculated as a single point at COSMO geometries. The systematic error of SM12 was corrected by −MSE. (**d**) Comparison between COSMO and COSMO-RS solvation models. The systematic error of COSMO-RS was corrected by −MSE.

In Figure 1, we report the scatter plots of computed vs. experimental redox potentials comparing different levels of approximation. The mean absolute error (MAE) and mean signed error (MSE) of each method are reported in the legends. In Figure 1a, we observe that gas-phase electronic energies, neglecting solvation and thermal contributions, yielded a rather bad prediction of experimental potentials: data were broadly scattered, and there was a significant systematic error. Including the solvation contribution improved the agreement (MAE ~ 0.3 V) and geometry optimization with a solvation model gave slightly better results than in the gas phase. The addition of the thermal contribution appears crucial for the prediction of redox potentials: the MAE was significantly reduced to 74 mV and the MSE was very small (6 mV), a negligible systematic error. This method (B3LYP optimization in COSMO + BLYP thermal correction) is the standard against which we will compare other methods. Figure 1b shows that computing the frequencies in gas or in solution had little effect on the thermal contribution, with a slight preference for the gas phase. We then compared two other solvation methods with COSMO. In Figure 1c, we show that with SM12 [38] single-point calculations (optimization is not possible in ADF) at COSMO geometries, we obtained a systematic error of 0.1 V. When empirically correcting for this error by subtracting the MSE from the computed potentials, the MAE became comparable with COSMO. In Figure 1d, we assess the performance of the COSMO-RS method, [39,40] which combines quantum chemistry with statistical mechanics. The solvation free energy obtained with COSMO-RS was added to the gas-phase energy. In this case, we also observe a systematic error of about 0.1 V, and when correcting for it, the MAE becomes almost identical to that of COSMO.

#### 2.2.3. Comparing Electronic Structure Methods

**Figure 2:**
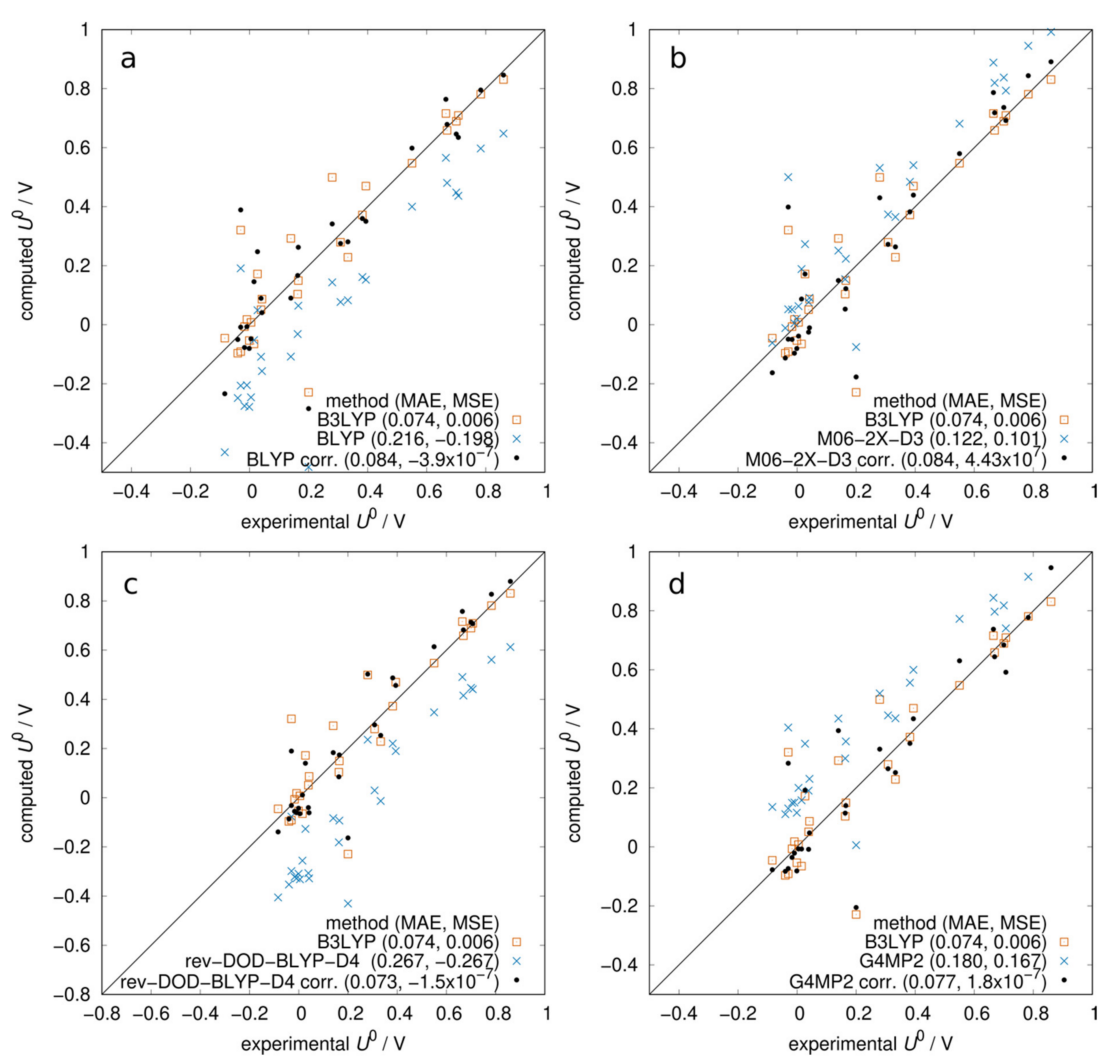
Computed vs. experimental redox potentials at pH = 0. Comparison between B3LYP and other methods. Systematic errors are corrected by subtracting MSE from all data points. All potentials include BLYP thermal correction except (**b**). (**a**) BLYP/ADZP optimized in COSMO. (**b**) M06-2X-D3(0)/6-31G(2df,p) optimized in COSMO with thermal correction obtained at the same level of theory in gas. (**c**) rev-DOD-BLYP-D4/TZ2P in COSMO (single point at B3LYP geometries). (**d**) G4MP2 gas-phase energies + B3LYP/COSMO solvation free energies.

Once the best set of approximations to the free energy contributions were determined, we turned our attention to the electronic structure method. While B3LYP is probably the most used off-the-shelf density functional for organic molecules, its performance in benchmark studies of molecular properties does not justify its popularity. In a recent benchmark study [41] performed with ADF, its performance for general properties and reaction energies (Figure 3 in [41]) was reported to be mediocre. Therefore, we consider it useful to compare B3LYP with a few different electronic structure methods: the GGA functional BLYP and two of the best performing functionals according to [41]: the meta-hybrid M06-2X-D3(0) and the double hybrid rev-DOD-BLYP-D4. We also recalculated the gas phase electronic energies using the composite method G4MP2 implemented in the Gaussian 16 software [42]. Both the double hybrid and G4MP2 included an energy term obtained with the second-order Møller–Plesset perturbation theory (MP2). As expected, BLYP performed worse than B3LYP (Figure 2a) even when correcting for the −0.2 V systematic error. The potentials obtained with M06-2X-D3(0) and 6-31G(2df,p) basis set with COSMO using Q-Chem 5.2 [43] (with thermal contribution obtained in the gas phase at the same level of theory) were affected by a +0.1 V systematic error. When correcting for that, the prediction was still slightly worse than B3LYP. With the double hybrid rev-DOD-BLYP-D4 (Figure 2c), we observed a negative systematic error and, after correcting for it, the MAE was not better than B3LYP. Similarly, Figure 2d shows that computing the electronic energies with the G4MP2 composite method and adding the B3LYP solvation free energy yields a systematic error (MSE = 0.17 V); when corrected, the method was not better than B3LYP.

Despite expectations, the B3LYP functional in conjunction with COSMO implicit solvation model appears to deliver the most accurate prediction of redox potentials among the several methods we tested. Since this property is essentially an energy difference, we can attribute the small MAE and MSE achieved by this method to fortuitous error cancellations. It should be noted that the choices of the two constants that enter Equation (1), $U_{\mathrm{SHE}}$ and $\Delta G_{s}^{0}\left(H^{+}\right)$, obviously affect all data points by the same amount. The discussion about the correct values of these constants can be found elsewhere, [44,45,46], and it is not clear which values are consistent with the COSMO model, but we note that with the values chosen here, the B3LYP/COSMO method had a reasonably good predicting power without the need for empirical correction of systematic errors.

#### 2.2.4. Limits of Implicit Solvation Models

Now we turn our attention to the outliers. For some of the molecules in the data set, the calculated potential is as much as ~0.4 V away from the experimental value. From the method comparisons in Figure 1 and Figure 2, we observe that these outliers are not affected by the choice of solvation model or electronic structure method. Therefore, we suspect that these large errors are due to the failure of implicit solvation models to capture specific interactions between molecule and solvent, which appear to have a large impact in isolated cases. This hypothesis can be verified by computing solvation free energies of these outliers with explicit solvent molecules. Since explicit solvation methods are time-consuming (both human and computational time) they are not suitable for high-throughput screening. Nevertheless, we select the outlier AQTH14 (for which the redox potential at pH = 0 is underestimated by ~0.4 V) to verify if computing the solvation free energy in the presence of explicit water molecules improves the agreement with the experimental potential. We adopt a cluster–continuum model as described by Bryantsev et al. [47] and compute the solvation free energies of the oxidized and reduced forms using the monomer cycle as described in ref. [47]. Under this approximation, the cluster–continuum (cc) solvation free energy of a solute A solvated by *n* water molecules is

(3) $$\Delta G_{s}^{\mathrm{cc}}\left(A\right)=\Delta G_{\mathrm{gas}}^{\mathrm{bind}}+\Delta G_{s}\left(\left[A{\left(H_{2}O\right)}_{n}\right]\right)+n\Delta G_{\mathrm{vap}}\left(H_{2}O\right).$$

$\Delta G_{\mathrm{gas}}^{\mathrm{bind}}$ is the binding energy of the cluster in the gas phase, calculated as the difference between the energies of the cluster $\left[A{\left(H_{2}O\right)}_{n}\right]$ and of its isolated components (A + *n*H_2_O). $\Delta G_{s}\left(\left[A{\left(H_{2}O\right)}_{n}\right]\right)$ is the solvation free energy of the cluster, i.e., the difference between its gas-phase and COSMO energies (both optimized in the respective phase). The last term is the vaporization free energy

(4) $$\Delta G_{\mathrm{vap}}\left(H_{2}O\right)=-\Delta G_{s}\left(H_{2}O\right)-RT\ln\left(55.34\right)-RT\ln\left(24.46\right),$$

where the first term is the COSMO solvation free energy of one water molecule in water, the second term is the free-energy change of one mole of water from 55.34 M (concentration of H_2_O in water) to 1 M, and the third is the free-energy change of one mole of an ideal gas from 1 atm to 1 M. The latter two corrections ensure that all reactants and products in gas phase and in solution are in the same 1 M standard state, as explained in ref. [47]. The free energies $G_{\mathrm{sol}}^{0}\left(\mathrm{Red},\mathrm{Ox}\right)$ in Equation (2) are then obtained as $G_{\mathrm{sol}}^{0}\left(A\right)=G_{\mathrm{gas}}^{0}\left(A\right)+\Delta G_{s}^{\mathrm{cc}}\left(A\right)$ where $G_{\mathrm{gas}}^{0}\left(A\right)$ is the sum of the B3LYP gas-phase energy and the BLYP thermal contribution.

**Figure 3:**
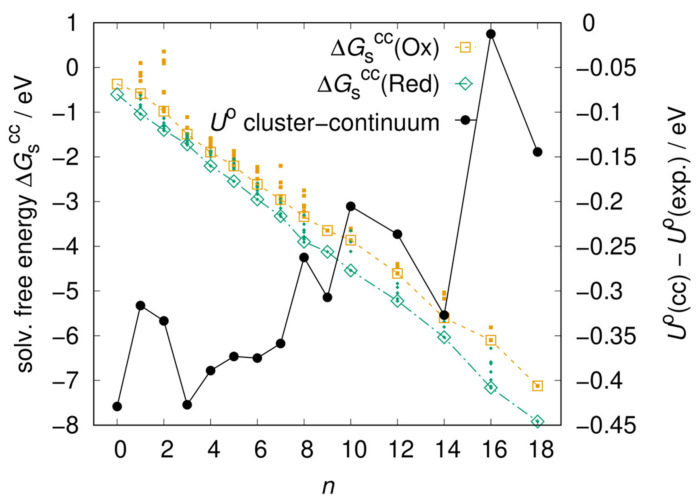
Left vertical axis: Solvation free energies of the Ox and Red forms of AQTH14 obtained from the cluster–continuum model as a function of the number *n* of explicit water molecules. Large empty symbols are the Boltzmann average over a few cluster conformations (small filled symbols). Right vertical axis: resulting standard redox potential expressed as the distance from the experimental value (0.200 V).

The literature on cluster–continuum models prescribes [47,48] that *n* should be increased until the solvation free energy $\Delta G_{s}^{\mathrm{cc}}\left(A\right)$ converges to a plateau. We manually built several clusters formed by the solute and *n* water molecules, with the goal of maximizing the number of solute–solvent and solvent–solvent hydrogen bonds. For each value of *n*, we pre-optimized several cluster geometries using the GFN-xTB semi-empirical method [49], optimized the most stable ones with DFT and then performed a Boltzmann average of $\Delta G_{s}^{\mathrm{cc}}\left(A\right)$ over the different clusters. We built clusters for *n* = 1–9, 10, 12, 14, 16, and 18. The clusters with *n* = 1–9 were built by placing water molecules on only one side of the molecular plane, and those with *n* = 10–18 were built by repeating the clusters with *n* = 5–9 on the other side of the molecular plane, taking advantage of symmetry. Only one cluster was considered for *n* = 9, 18 respectively since nine waters were found to form a stable 3 by 3 cluster, and no other stable geometries were found. As shown in Figure 3, the solvation free energies of both redox forms decrease when increasing *n* thanks to the solvating effect of the explicit water molecules, but none of them has converged to a plateau yet. The resulting redox potential becomes closer to the experimental value, although not linearly with *n*, confirming our hypothesis that the error is due to specific solute-solvent interactions missed by implicit solvation (*n* = 0). The non-linear behavior may be due to the fact that the manually built and optimized clusters do not constitute a sample large enough to achieve a converged average. We anticipate that better sampling and convergence of the solvation free energy to a minimum could be achieved by extracting solute-solvent clusters from a molecular dynamics simulation at finite temperature, but such undertaking is beyond the scope of this study.

### 2.3. Transformation to Higher pH Values

When electron transfer is coupled to proton transfer, the redox potential depends on the pH. The transformation of the pH = 0 redox potential to higher pH values can be achieved by modifying the free energies of Red and Ox according to the Alberty-Legendre transform [21,50]:

(5) $$G_{\mathrm{sol}}^{\mathrm{pH}}=G_{\mathrm{sol}}^{0}+N_{H}RTln\left(10\right)\mathrm{pH},$$

where $G_{\mathrm{sol}}^{0}$ is the free energy at pH = 0, $N_{H}$ is the number of hydrogen atoms at pH = 0, *R* is the Rydberg constant, and *T* is the temperature. In contrast to Equations (4)–(10) in [50], here we neglect the dependence on the ionic strength. At a given pH, the redox potential *U* is directly proportional to the free energy difference $\Delta G_{\mathrm{sol}}^{\mathrm{pH}}$ and the slope of the *U* vs. pH curve at 298.15 K will therefore be $RTln\left(10\right)=0.059\;V$, multiplied by the number of protons that are transferred to the molecule at pH 0 $\left(N_{H}\left(\mathrm{Red}\right)-N_{H}\left(\mathrm{Ox}\right)\right)$. This approximation, under which the slope is constant and independent of pH, neglects the fact that the redox species will be deprotonated at pH > ${\mathrm{pK}}_{a}$.

We have refined this method by updating the slopes of $G_{\mathrm{sol}}^{\mathrm{pH}}\left(\mathrm{Red}\right)$ and $G_{\mathrm{sol}}^{\mathrm{pH}}\left(\mathrm{Ox}\right)$ at each of the ${\mathrm{pK}}_{a}$ values obtained from ChemAxon [22]. In practice, we construct approximate Pourbaix diagrams for $G_{\mathrm{sol}}^{\mathrm{pH}}\left(\mathrm{Red}\right)$ and $G_{\mathrm{sol}}^{\mathrm{pH}}\left(\mathrm{Ox}\right)$ and consequently for $U^{\mathrm{pH}}$. For each redox form, we evaluate $G_{\mathrm{sol}}^{{\mathrm{pH}}_{p}}$ for a set of pH values ${\mathrm{pH}}_{p}=\left\{0,\ldots ,{\mathrm{pK}}_{a1},\ldots ,{\mathrm{pK}}_{a2},\ldots ,14\right\}$ which includes any ${\mathrm{pK}}_{a}$ values in the range (0, 14). $G_{\mathrm{sol}}^{0}$ is given by Equation (2), and for ${\mathrm{pH}}_{p}$ > 0 we use the formula

(6) $$G_{\mathrm{sol}}^{{\mathrm{pH}}_{p}}=G_{\mathrm{sol}}^{{\mathrm{pH}}_{p-1}}+N_{H}^{{\mathrm{pH}}_{\mathrm{half}}}RTln\left(10\right)\left({\mathrm{pH}}_{p}-{\mathrm{pH}}_{p-1}\right).$$

The number of hydrogens is that of the major microspecies predicted by ChemAxon at a pH value ${\mathrm{pH}}_{\mathrm{half}}={\;\mathrm{pH}}_{p-1}+1/2\left({\mathrm{pH}}_{p}-{\mathrm{pH}}_{p-1}\right)$, i.e., halfway between the current and the previous. This ensures that the slope of the segment ${\mathrm{pH}}_{p-1}-{\mathrm{pH}}_{p}$ always depends on the molecule’s updated protonation state, including cases where ${\mathrm{pH}}_{p-1}$ is one of the ${\mathrm{pK}}_{a}$s.

**Figure 4:**
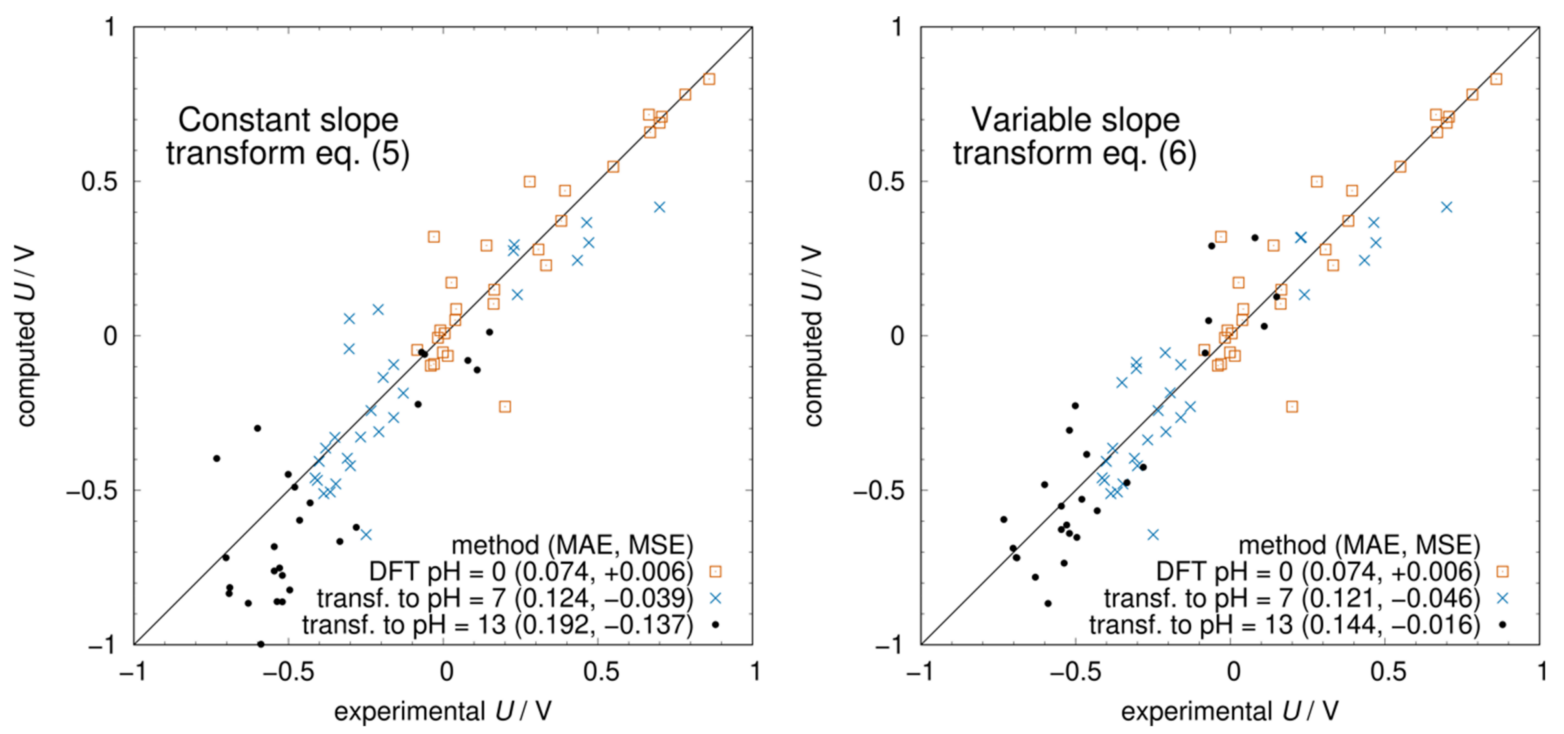
Computed vs. experimental redox potentials at different pH values. In the (**left panel**), the transformation from pH 0 to 7 and 13 was carried out with Equation (5) using the number of protons at pH = 0. In the (**right panel**), the transformation is done with Equation (6) where the number of protons and hence the slope of the Pourbaix diagram was updated at every ${\mathrm{pK}}_{a}$.

The free energies computed with either Equation (5) or (6) enter Equation (2), and the potential as a function of pH is then obtained from Equation (1). The resulting Pourbaix diagrams of all molecules are shown in Figure S1 in the Supplementary Materials. In Figure 4, we compare the experimental potentials with those computed at pH = 0 and those transformed to pH = 7 and 13 using Equations (5) and (6). At pH = 7, there is not much difference between the two methods because almost all molecules are in the same protonation state as at pH = 0, i.e., there are no ${\mathrm{pK}}_{a}$s below 7. On the other hand, at pH = 13, most molecules have undergone some deprotonation leading to a smaller slope of the *U* vs. pH curve. As a result, using Equation (5) leads to a systematic underestimation of the potentials (MSE = −0.137 V). The adjustment of the slope using Equation (6) corrects this systematic error (MSE = −0.016 V) and significantly improves the agreement with the experimental potentials. Considering the low computational cost of obtaining the ${\mathrm{pK}}_{a}$s and protonation states using ChemAxon, the proposed procedure to transform pH = 0 potentials using Equation (6) should become a standard method for predicting redox potentials at any pH value.

It is, of course, also possible, instead of transforming the pH = 0 potentials, to compute the pH = 7 and 13 potentials directly with DFT using the same procedure as for the pH = 0 potential: (1) Determine the major protonation state at a given pH using ChemAxon; (2) Find lowest energy conformer; (3) Compute free energies of Ox and Red to determine the redox potential with Equations (1) and (2). Since most molecules in our data set have the same protonation state at pH = 0 and 7, we only report the potentials obtained directly at pH = 13. As shown in Figure S2 (Supplementary Materials), this method leads to a large underestimation of the redox potentials, that we attribute to the consistent failure of the implicit solvation model to capture the full solvation free energies, especially of the reduced forms, which have larger negative charges. This result confirms that, as discussed earlier, it is much more accurate to compute free energies at pH = 0 protonation states and then transform them to the pH of interest.

## 3. Summary and Conclusions

In this contribution, we have benchmarked a procedure to predict redox potentials of organic molecules from the first principles. Taking as the input the SMILES strings of the reduced and oxidized forms, we show that widely available cheminformatics tools can be used to determine the main protonation state at pH = 0 and the major conformer. Two DFT calculations for each redox form are then needed to compute the redox potential: one geometry optimization with COSMO implicit solvation and one geometry optimization with subsequent frequency calculation in the gas phase. The former includes a reasonable estimate of the solvation free energy, and the latter provides an estimate of the thermal contribution to the free energy. Among several electronic structure methods tested (including composite methods), the B3LYP hybrid density functional was found to yield the best agreement with a consistent set of experimental potentials (MAE = 0.074 V and MSE = −0.006 V without empirical corrections). It may seem surprising that B3LYP, which is the most popular but not particularly new or generally accurate functional, performs better for redox potentials than other methods reported to perform best in other more comprehensive benchmarks. The very popular combination of B3LYP with double-ζ polarized basis sets (6-31G* or similarly ADZP) is believed to be the cause of the error cancellations (errors due to the functional and to the basis set incompleteness are similar and of opposite sign) [51] that make it reasonably accurate in many cases, especially when including a dispersion correction such as D3 [52].

In a few cases, the potential prediction was found to be up to 0.4 V off the experimental value. By employing a mixed explicit–implicit solvation model on one of these outliers, we have verified that such errors are very likely due to the failure of the implicit solvation model to capture specific solvent-solute interactions. These exceptional cases cannot be predicted from the outset. In the near future, significantly more effort should be dedicated to the establishment of rapid protocols for computing reasonably good solvation free energies with explicit solvation. Such protocols that overcome the limitations of implicit solvation could then be used in high-throughput screening studies where a more accurate prediction of redox potentials is desirable.

Finally, we presented a novel and computationally inexpensive procedure to transform the pH = 0 redox potentials computed with DFT to any other pH value. Using the ${\mathrm{pK}}_{a}$s predicted by the ChemAxon cheminformatics software [22], we construct an accurate estimation of the Pourbaix diagram that takes into account the changes in slope of the *U* vs. pH curve due to deprotonations of both redox forms. The potential predictions at pH = 13 obtained with this variable slope approach correct the systematic underestimation resulting from the constant slope transformation that considered only the pH = 0 protonation states. The MAE with respect to experimental potentials were found to be 0.121 V at pH = 7 and 0.144 V at pH = 13 with our new method.

**Scheme 2:**
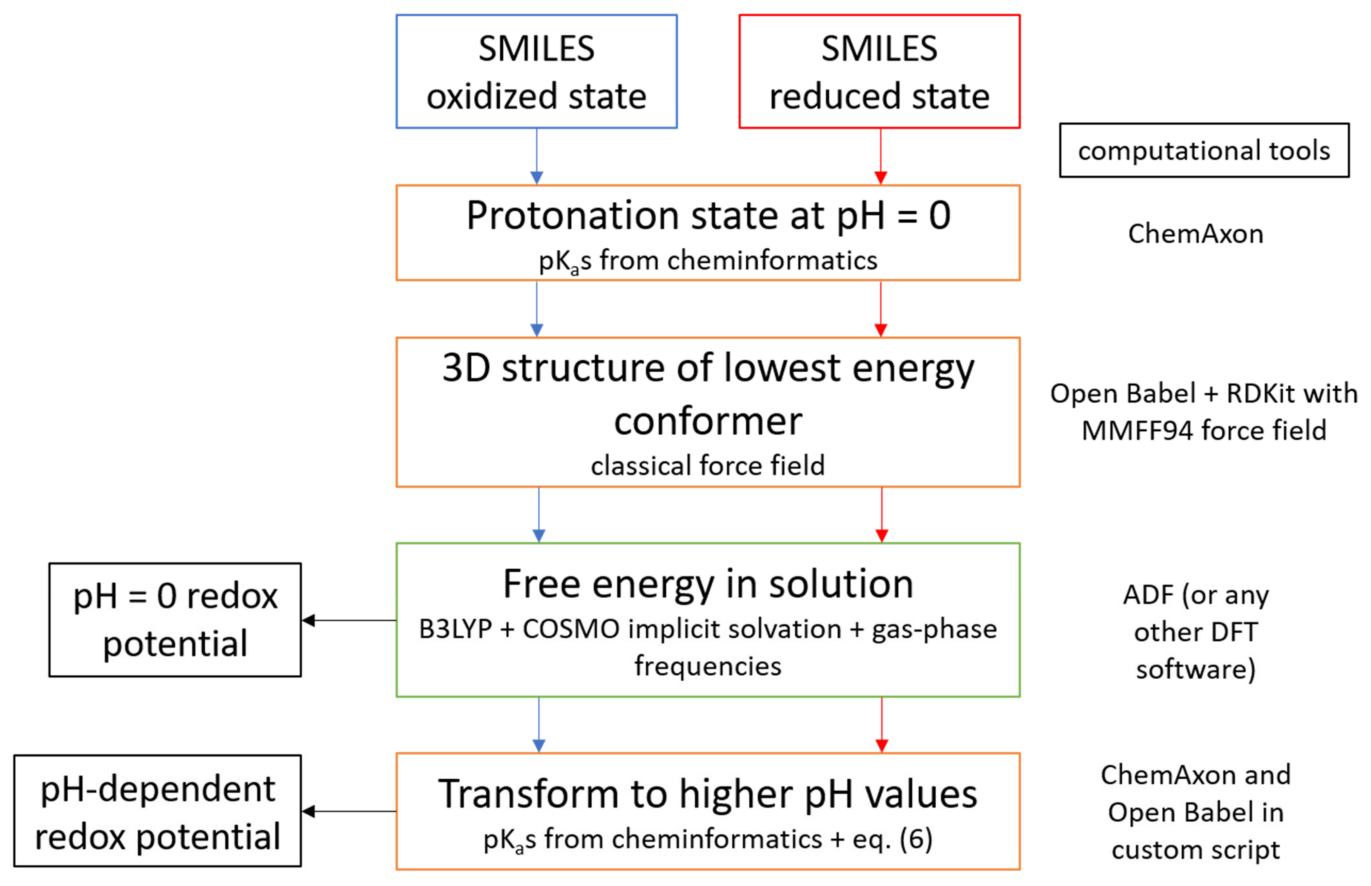
Flowchart of the computational protocol described in this article.

In summary, we have established and validated a protocol to predict redox potentials of organic molecules at a modest computational cost, using standard DFT methods and widely available cheminformatics tools that can be easily implemented in high-throughput screening studies. Scheme 2 illustrates all steps of the procedure and the computational tools necessary for each step. We have shown that with this protocol, redox potentials can be predicted with MAE below 0.15 V over a wide range of pH.

## Data Availability

The data presented in this study are openly available in FigShare at https://doi.org/10.6084/m9.figshare.14866749, accessed on 7 June 2021.

## Supplementary Materials

The following are available online, Table S1: Full names of all molecules. Figure S1: Pourbaix diagrams (redox potential vs. pH) of all molecules, Figure S2: Computed vs. experimental redox potentials computed directly at pH = 13.
