# Supplementary material for: A Computational Protocol Combining DFT and Cheminformatics for Prediction of pH-Dependent Redox Potentials
Source: Molecules. 2021 Jun 29;26(13):3978. doi: 10.3390/molecules26133978 (PMC8271517; doi:10.3390/molecules26133978)
Supplement: Supplementary file 1 [file molecules-26-03978-s001.zip › molecules-1271786-supplementary.pdf]

Supplementary Materials:
A computational protocol combining DFT and cheminformatics for prediction of pH-dependent redox potentials

Rocco Peter Fornari [1] and Piotr de Silva [1,*]

[1] Department of Energy Conversion and Storage, Technical University of Denmark, Anker Engelunds Vej 301, 2800 Kongens Lyngby, Denmark

* Correspondence: pdes@dtu.dk

Table S1. Full chemical names of the molecules considered.

Abbreviation	(common/commercial name): full name
AQDH12	(Alizarin): 1,2-dihydroxy-9,10-anthraquinone
AQDH14	(Leucoquinizarin): 2,3-dihydro-9,10-dihydroxy-1,4-anthraquinone
AQDH15	(Anthrarufin): 1,5-dihydroxy-9,10-anthraquinone
AQDH18	(Chrysazin): 1,8-dihydroxy-9,10-anthraquinone
AQDH26	(Anthraflavic acid): 2,6-dihydroxy-9,10-anthraquinone
AQDS27	9,10-anthraquinone-2,7-disulfonate
AQDS15	9,10-anthraquinone-1,5-disulfonate
AQDS18	9,10-anthraquinone-1,8-disulfonate
AQS2	9,10-anthraquinone-2-sulfonate
AQS2DH	(Alizarin red s): 3,4-dihydroxy-9,10-anthraquinone-2-sulfonate
AQS2NBr	1-amino-4-bromo-9,10-anthraquinone-2-sulfonate
AQDH45CA	(Rhein): 4,5-dihydroxy-9,10-anthraquinone-2-carboxylic acid
AQDH18MH	(Aloemodin): 1,8-dihydroxy-3-(hydroxymethyl)-9,10-anthraquinone
AQTrHM	(Emodin): 1,3,8-trihydroxy-6-methylanthracene-9,10-dione
AQTH12	(Quinalizarin): 1,2,5,8-tetrahydroxy-9,10-anthraquinone
AQTH14	1,4,5,8-tetrahydroxy-9,10-anthraquinone
NQ12S	1,2-naphtoquinone-4-sulfonate
NQ14HB	(Lapachol): 2-hydroxy-3-(3-methyl-2-butenyl)-1,4-naphthoquinone
NQ14H	2-hydroxy-1,4-naphtoquinone
HQ14S	2,5-dihydroxybenzenesulfonate
HQ12	Ortho hydroquinone
HQ14	Para hydroquinone
HQ12DS	(Tiron): 4,5-dihydroxy-1,3-benzenedisulfonate
BQ14DH	2,5-dihydroxy-1,4-benzoquinone
BQ14DHDCl	(Chloranilic acid): 2,5-dichloro-3,6-1,4-benzoquinone
HQ14TCl	Tetrachloro hydroquinone
BQ14TH	Tetrahydroxy-1,4-benzoquinone
HQ14TF	1,2,4,5-tetrafluoro-3,6-dihydroxybenzene

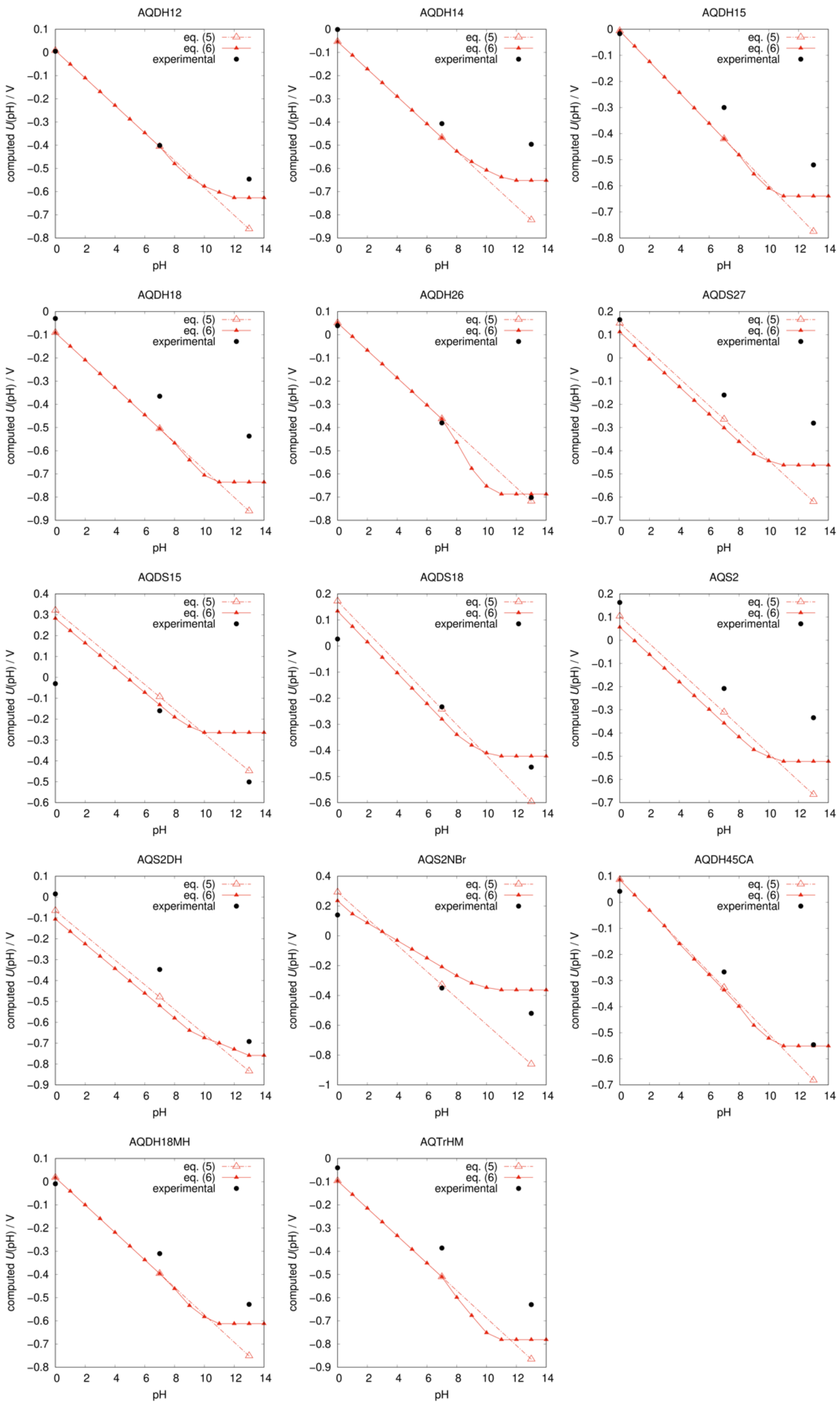
AQDH12
AQDH14
AQDH15
AQDH18
AQDH26
AQDS27
AQDS15
AQDS18
AQS2
AQS2DH
AQS2NBr
AQDH45CA
AQDH18MH
AQTrHM
eq. (5)
eq. (6)
experimental
computed U(pH) / V
pH

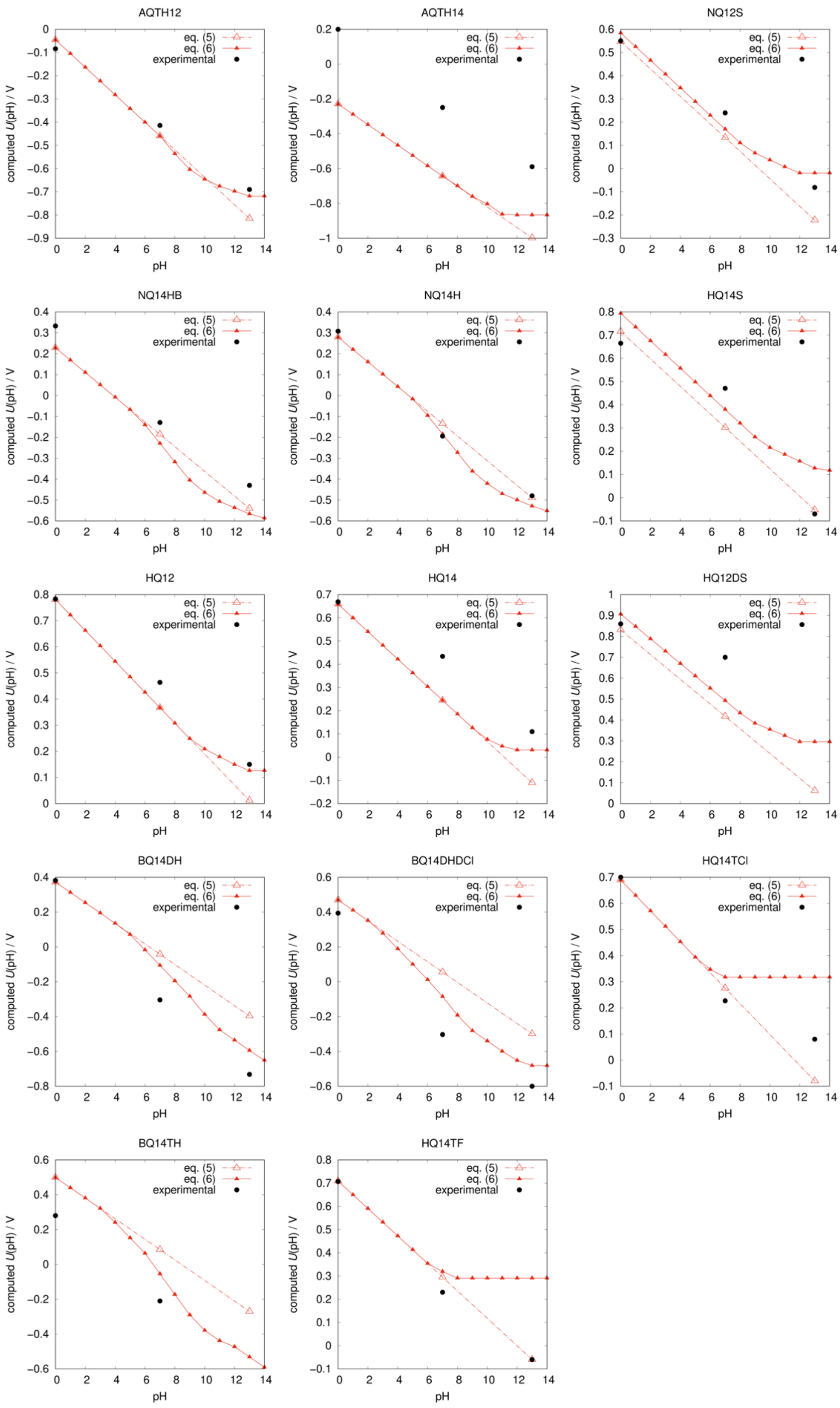

Figure S1. Pourbaix diagrams (potential vs pH) of all molecules, built with eqs. (5) and (6) (main text). Experimental potentials from ref. [17] are also shown.

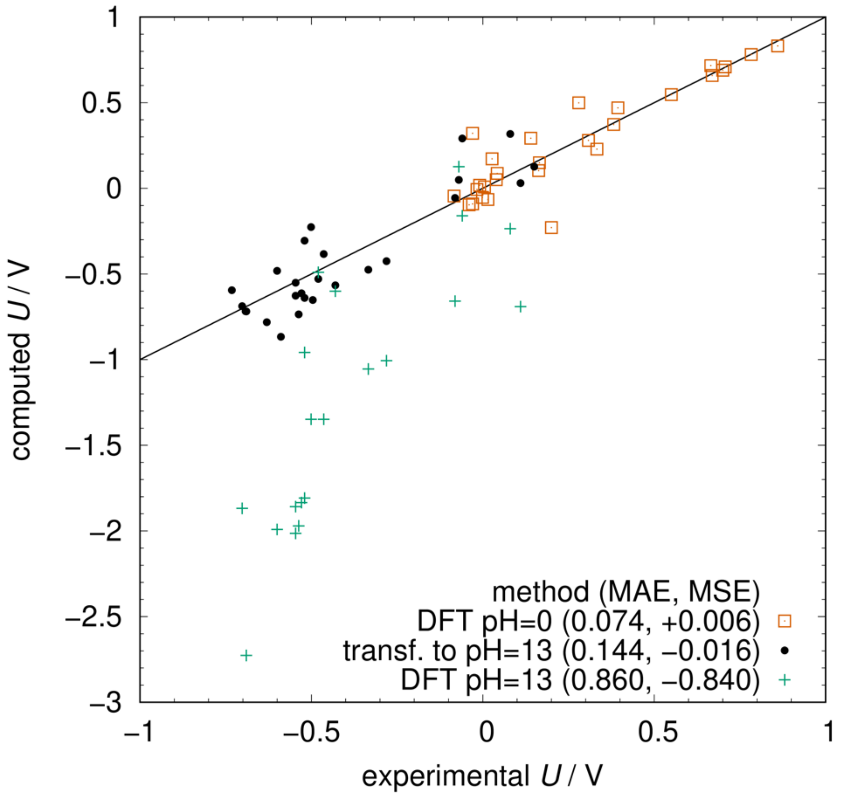

Figure S2. Computed vs. experimental redox potentials computed directly with DFT at pH=0 and 13, and transformed to pH 13. The DFT results at pH=13 are obtained by optimizing the molecular geometries in the pH=13 protonation state predicted by ChemAxon. The other data sets are discussed in the main text.
